# Room in the Profession—Letter and Answer

**Published:** 1868-09

**Authors:** 


### Editorial Department.

### Room in the Profession—Letter and Answer.

We publish the following communication and brief reply, as this is an inquiry made quite often in one form and another. Our reply, when published, will answer them all, so far as the question of "room" and "great inducements" require answer:

RUSHVILLE, September 2, 1868.

To the Editor of the Buffalo Medical and Surgical Journal:

My Dear Sir:—I write to inquire of you if there is yet room in the medical profession for my son? He has been well educated in the primary branches and in the languages, and seems to have a preference for medicine rather than law. I have no desire to dictate his course, but would advise him according to my best judgment after being myself well informed. I would like to know if there are great inducements for an active young man to study medicine, or could he turn his attention to law or trade with better prospects of success? You will excuse the liberty I have taken, when you remember your old patient, and truly your friend, J. M. CASH.

P. S.—When do the Lectures commence in the Buffalo Medical College, and should Richard conclude to study medicine, will it be as well to commence with this course? J. M. C.

BUFFALO, September 12, 1868.

Mr. Cash—My Dear Sir:—Your letter has remained on my table unanswered for some days, as I could hardly make up my mind what to say in reply to the main question of your letter, "Is there any room in the medical profession for my son?" *Yes*, would answer your question truly and briefly, but you would not be satisfied with such brevity, and I must tell you what and where the room is, and how your son can fill it. Do you want to know if there are large towns and villages without resident physicians, waiting for your son to graduate and commence the practice of medicine? Large towns and villages have many physicians; better for the physicians and towns if there were less. One physician to every four or five hundred inhabitants throughout our part of the world, to say nothing of the numberless Doctor's signs, put up often in old-fashion hotel style, minus one post, where all the Doctor there is, is *on the sign*, is about the room, in our country, for a physician. You will not infer from this, that I think there is no room for your son, for, my dear sir, there will always be a great vacuum for the medical profession to fill, and your son might be drawn into it, and a great many more such boys, and still it would be as large as ever. This room is wholly unoccupied in a great majority of the finest towns and villages of the world, and it offers at the present time the very highest attractions—everything which a young man may truly prize is here within his grasp, but it is "*high up*"—is above all these signs of which I have spoken, and above most of those who make the one physician to every five hundred inhabitants, above all who practice medicine as a trade; it is so high that it will take considerable time and much climbing to reach it, and during all the earlier years of study and labor there will be little if any profits, speaking after a worldly manner. There is little money to be made out of medicine in a short time, and if you mean to inquire if there is room to make money in the profession of medicine, I should say yes, unconditionally, all the room is yet left for your son; no one I have ever known in the profession, has yet occupied it. There is room then in *every* city and town, and your inquiry is answered yes—room for study, for thought, for investigation, for practice, and if you care to know it, room for more money than will ever be obtained. If your son is ambitious and in earnest to make a physician of himself, ready to devote to it his whole energy and thought; is not wavering or fickle in his purpose, but ready to sacrifice to *medicine alone*, there is a wide field full of promise, and I earnestly invite him to commence a business which is exhaustless in its extent, attractive in the pursuit, and noble and humane in its objects and aims.

Lectures commence November 4th, and I shall be glad to see your son in attendance; there is no better or surer way to early obtain the true medical inspiration than by attending lectures.

Yours, very truly, J. F. MINER.

